# Change in Contraceptive Use Within South Carolina Medicaid Following the Choose Well Contraceptive Access Initiative: Did COVID-19 Alter the Trends?

**DOI:** 10.1089/whr.2024.0040

**Published:** 2024-09-24

**Authors:** Wondi Samuel Manalew, Nathan Hale, Michael G. Smith, Amal J. Khoury

**Affiliations:** Department of Health Services Management and Policy, Center for Applied Research and Evaluation in Women’s Health, East Tennessee State University, Johnson City, TN, USA.

**Keywords:** South Carolina, Medicaid women, contraception, Choose Well, COVID-19, interrupted time-series analysis

## Abstract

**Objectives:**

COVID-19 hit at the midpoint of Choose Well, a statewide contraceptive access initiative commenced in South Carolina (SC) in 2017. This study assessed whether the pandemic altered the trends in contraceptive use among SC Medicaid during the first half of Choose Well.

**Methods:**

Contraception use among 333,253 women was analyzed from 2017 to 2022, divided into *prepandemic* (January 2017–February 2020) and *pandemic* (March 2020–December 2022) periods. Bivariate differences in contraceptive use were examined using Pearson’s Chi square test across these periods, including *the first*, *first two*, and *first three* quarters of the pandemic. Interrupted time-series analysis assessed changes in trends for intrauterine devices (IUDs) and implants *during pandemic* compared with *prepandemic* levels.

**Results:**

IUD and implant use dropped during the first two quarters of the pandemic. While IUD use matched the prepandemic levels by the end of the first three quarters, implant use slightly lagged. The use of injections and pills decreased from 16.6% and 26.2% during the *prepandemic period* to 13.6% and 21.7% during the *pandemic period*, respectively (*p* < 0.001). The trends in IUD and implant use in the pandemic period were higher by 0.01 (95% confidence interval [CI]: 0.01, 0.02) and 0.04 (95% CI: 0.03, 0.05) percentage points per month relative to the prepandemic trends, respectively.

**Conclusions:**

The pandemic’s initial impact quickly stabilized, and overall, the gains in contraceptive use among Medicaid beneficiaries associated with Choose Well remained largely unaffected, with some methods showing increased trends.

## Introduction

The Choose Well contraceptive access initiative began in South Carolina (SC) in 2017 as the first and largest initiative of its kind in the US Southeast. The statewide initiative aimed to promote equitable access to contraception among population groups and reduce unintended pregnancy by 2023. Initially envisioned as a 6-year initiative, Choose Well is engaged a wide range of healthcare organizations across the state, providing cost coverage for contraceptive methods. In addition, Choose Well engaged in workforce and infrastructure development, capacity building and training, and marketing and communications efforts aimed at enhancing contraceptive access across the state, particularly for low-income women. Central components of the Choose Well initiative have been described elsewhere.^1^

Crossing the midpoint of the evaluation, early evidence indicates that Choose Well has been successful in fostering access to a wide range of contraceptive methods. External evaluation studies of Choose Well have shown that the statewide training offered by the initiative was effective in enabling family planning staff and providers to enhance their contraceptive practices. These trainings also increased the reported clinical capacity for offering contraceptive services, especially among Federally Qualified Health Centers (FQHCs) in SC.^2,3^ Interventions to improve access to contraceptives translated to increased stockpiling and provision of the full range of contraceptive methods among FQHCs^4^ and long-acting reversible contraceptive method use among women enrolled in the state’s Medicaid program increased notably following Choose Well.^5^ These evaluation studies reflect the midpoint of the evaluation, which occurred prior to the onset of the COVID-19 pandemic.

Three years into the Choose Well initiative, the national landscape was abruptly reshaped by the onset of the pandemic. COVID-19-related challenges beyond the direct consequences of the infection disrupted the ability to provide many health services, creating significant barriers to care.^6^ During different phases of the COVID-19 pandemic, hospitals, clinics, and state governments temporarily halted nonessential health services to prioritize the care of COVID-19 patients.^7^ While hospitals operated at capacity due to surges in COVID-19 throughout various stages of the pandemic, utilization of healthcare services unrelated to COVID-19 decreased.^8^ Studies have noted that access to contraception became increasingly difficult during the pandemic, citing travel restrictions, quarantine measures, additional caregiving responsibilities, fear of exposure to the virus, and fewer available appointments as barriers to receipt of contraceptive services.^9^

This study examines the impact of the pandemic on contraceptive method utilization among women enrolled in the SC Medicaid program. The onset of the pandemic has significant implications for the evaluation of Choose Well and for measuring the impact of the initiative on receipt of contraceptive services and subsequent outcomes within the state. Previous research examining the impact of Choose Well leading up to the pandemic noted that the use of intrauterine devices (IUDs) and implants among Medicaid beneficiaries increased at the onset of Choose Well and that monthly trends in IUD use during the Choose Well intervention period between January 2017 and February of 2020 were above what would have been expected in the absence of Choose Well.^5^ The extent to which the pandemic disrupted access to contraceptive methods and potentially altered utilization patterns associated with Choose Well implementation within the state remains unknown. Beyond the implications for the Choose Well evaluation, state Medicaid programs remain pivotal in fostering access to care for under-resourced persons with increased vulnerabilities. As such, this study is also important for understanding the extent to which Medicaid enrollees experienced disruptions in services caused by the pandemic.

## Materials and Methods

### Data and study design

For this study, a retrospective cohort of women of reproductive age (15–45) enrolled in SC Medicaid between January 2017 and December 2022 was created, covering a period of roughly 3 years before and 3 years after the onset of the COVID-19 pandemic. Medicaid eligibility and claims data were acquired through data use agreements with the SC Office of Revenue and Fiscal Affairs. Approval from the Institutional Review Board was granted by East Tennessee State University. Examining the impact of the COVID-19 pandemic on contraceptive use trends during the second half of Choose Well relative to the prepandemic trend was of primary interest. Medical and pharmacy claims from Medicaid were utilized to identify contraception use and determine the types of contraceptive methods used. IUD service use, implant service use, IUD insertion, implant insertion, injection service use, and pill service use were examined. “IUD/implant service use” encompasses a comprehensive range of services related to IUDs/implants, including counseling, follow-up visits, and insertion, whereas “IUD/implant insertion” refers specifically to the initial placement of the devices. Due to the absence of distinct codes for new prescriptions for pills and injections in our administrative dataset, all services related to pills and injections were categorized as “pill service use” and “injection service use,” respectively.

Consistent with previous studies,^5,10^ the study population was restricted to women qualifying for full benefits, women enrolled in limited-benefit family planning, and teenage-dependent children with coverage through partners for healthy children programs. While this excluded some eligibility categories, these three programs accounted for 78.3% of all women enrolled over the study period and those most likely to engage in contraceptive use.

Since Medicaid data do not provide information on pregnancy intention or sexual activity, the study was further refined to focus on women who showed evidence of engaging in reproductive health services. This was identified through family planning claims, which included services such as contraceptive provision and counseling, emergency contraception, fertility awareness education, and sexually transmitted infection testing, treatment, or counseling. By focusing on these specific claims, the study narrowed its scope to women actively utilizing reproductive health services, ensuring the inclusion of those most likely to seek contraceptive care within the Medicaid program.

January 2017 marked the start of the Choose Well initiative in SC. A state of emergency was declared in March of 2020, and a series of containment policies were implemented both at the national level and in the state of SC, leading to widespread lockdowns and restrictions on movement. The initial duration of the Choose Well intervention period (2017–2022) was divided into two approximately equal periods: the *prepandemic period,* which represents the period from January 2017 to February 2020 (38 months), and the *pandemic period*, consisting of the period from March 2020 to December 2022 (34 months). To further explore the extent to which contraceptive method use changed in the immediate wake of the pandemic, the first year of the pandemic (March–December 2020) was divided into three periods: *the first quarter* (March 2020–May 2020), *the first two quarters* (March 2020–August 2020), and the *first three quarters* (March 2020–December 2020).

### Analysis

The demographic characteristics and the types of contraceptive methods in the study population were described by person years. The analysis included a three-level categorical variable for age groups: adolescents (15–19), young adults (20–25), and adults (26–45). Race and ethnicity were classified as non-Hispanic White, non-Hispanic Black, Hispanic, and non-Hispanic other.

The study investigated bivariate differences in contraceptive use, comparing the *prepandemic* and *pandemic* periods using Pearson’s Chi-Square test by person year. To assess how contraception patterns evolved over time among women enrolled in SC Medicaid during the pandemic, we separately compared contraceptive use in *the first quarter*, *the first two quarters*, and *the first three quarters* of the pandemic with contraceptive use in the prepandemic period using Pearson’s Chi square test.

An interrupted time-series analysis (ITSA) was used to examine whether the overall trend in contraceptive use during the *pandemic period* significantly differed from the trend observed in the *prepandemic period*. The ITSA evaluates trends in monthly contraceptive use and enables control for trends and seasonality in contraceptive method usage both prior and during the pandemic.^11^ The single-group ITSA regression model, which estimates treatment effects for a single interruption period, is represented as follows:

$$Y_{t}\;=\;\beta_{o}\;+\;\beta_{1}T_{t}\;+\;\beta_{2}P\mathrm{ost}\;+\;\beta_{3}(T_{t}-T_{i})\;\mathrm{Post}\;+\;\beta_{4}S_{t}\;+\;\varepsilon_{t}$$

where ${\text{Y}}_{\text{t}}$ represents the contraceptive method use at month t, ${\text{T}}_{\text{t}}$ is a continuous time variable (trend) capturing time elapsed since the start of the study period (January 2017), Post is a binary indicator for the pandemic period (0 for *prepandemic period* [January 2017–February 2020] otherwise 1 [March 2020–December 2022]), ${\text{T}}_{\text{i}}$ represents the time point when the pandemic started, and $(T_{t}-T_{i})\;\mathrm{Post}$ is an interaction between the pandemic dummy ( Post) and the time elapsed since the beginning of the pandemic ( $T_{t}-T_{i}$), and it captures the change in the trend in the pandemic period, ${\text{S}}_{\text{t}}$ is a quarterly dummy used to control for seasonality, and ${\text{ε}}_{\text{t}}$ is the error term. ${\text{β}}_{0}$ represents the intercept or starting level of the outcome (percentage of women choosing a method at the beginning of the study period), ${\text{β}}_{1}$ measures the average monthly change in method use over the prepandemic period, ${\text{β}}_{2}$ represents the change in the level of method use in the period immediately following the pandemic compared with method use that would be expected had the pandemic never occurred. ${\text{β}}_{3}$ represents the difference between prepandemic and pandemic period trends in method use and assesses the extent to which the pandemic influenced the method use beyond what would be expected. A statistically significant value in ${\text{β}}_{2}$ indicates an immediate pandemic effect (change in the level), and a statistically significant value in ${\text{β}}_{3}$ indicates an effect over time (the effect on trend).^12^

The ordinary least squares regression was used to conduct the ITSA. The regression analysis accounted for differences in age, race and ethnicity, and the Medicaid eligibility category of the women. To ensure that the model accurately accounted for the correct autocorrelation structure, an autocorrelation test was conducted. For models where autocorrelation was detected, Newey–West standard errors were applied to adjust for the autocorrelation.

## Results

### Study sample

The study population included 333,253 reproductive age women who were eligible for SC Medicaid benefits between 2017 and 2022. The individual women contributed to 2,420,998-person month observations and 916,575 person-years. Characteristics of the study population summarized by person-years are described in Table 1. Non-Hispanic White women and non-Hispanic Black women accounted for 72% of the person-years, whereas all other races including women of Hispanic ethnicity represented about 28% of the person-years. Approximately half of the women (48.6%) in the study population were 25 years old or younger, whereas women 26–45 years of age comprised the remaining 51.4% of the person-years (Table 1).

**Table 1.** Characteristics of Women of Reproductive Age Enrolled in South Carolina Medicaid and Contraceptive Method Use: Person-Years

	IUD service use *n* = 47,867 (5.2%)	IUD insertion *n* = 27,445 (3.0%)	Implant service use *n* = 52,548 (5.7%)	Implant insertion *n* = 30,599 (3.3%)	Injection service use *n* = 139,516 (15.2%)	Pill service use *n* = 221,130 (24.1%)	No evidence *n* = 464,791 (50.7%)	Total Total = 916,575
	%	%	%	%	%	%	%	*n* (%)
Race/Ethnicity								
Non-Hispanic White	6.2	3.68	5.18	3.11	10.26	29.68	49.18	287,058 (31.3)
Non-Hispanic Black	4.1	2.2	5.76	3.15	20.43	20.2	51	374,625 (40.9)
Non-Hispanic Other	5.83	3.4	6.22	3.81	13.77	23.41	51.7	208,648 (22.8)
Hispanic	4.82	2.86	6.25	4.19	6.62	21.6	60.55	22,453 (2.4)
Missing	6.17	3.88	7.2	4.06	13.92	27.59	46.67	23,791 (2.6)
Age groups								
Ages 15–19	2.61	1.9	6.09	4.23	16.31	32.49	45.96	259,864 (28.4)
Ages 20–25	7.16	4.06	8.84	4.59	17.72	27.69	40.58	185,544 (20.2)
Ages 26–45	5.9	3.18	4.31	2.36	13.64	18.11	57.32	471,167 (51.4)

Method totals are based on claims encountered for individual women. Individual women with claims for multiple methods may be counted more than once.

IUD, intrauterine device.

Short-acting hormonal methods, injections, and pills were the most common contraceptive methods used, with approximately 40.0% of women using these methods. IUDs and implants accounted for 5.2% and 5.7% of method use, respectively. Approximately 50% of the person-years were represented by women with no evidence of contraceptive claims. Higher percentages of IUD and implant use were observed among women in the 20–25 age group relative to the other age groups (Table 1).

### Changes in contraceptive use during the pandemic: Bivariate analysis

**FIG. 1.**
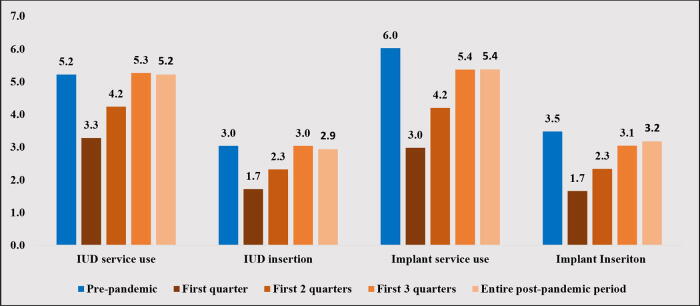
Changes in contraceptive method use after the COVID-19 pandemic among women enrolled in South Carolina Medicaid.

Changes in IUD and implant use following the onset of the COVID-19 pandemic are provided in Figure 1. IUD use significantly dropped during the first quarter of the pandemic compared with the prepandemic level (3.3% vs. 5.2%, *p* < 0.001). IUD use was still significantly lower than its prepandemic level during the first two quarters of the pandemic (4.2% vs. 5.2%, *p* < 0.001). By the third quarter, there was no statistical difference in the IUD use relative to the prepandemic period (5.3% vs. 5.2%, *p* > 0.05). IUD insertion during the pandemic followed a similar pattern. The use of implants substantially dropped from 6.0% before the pandemic to 3.0% during the first quarter of the pandemic (*p* < 0.001). Implant use reached 4.2% at the end of the first two quarters and 5.4% at the end of the first three quarters (*p* < 0.001). Implant insertion followed a similar path (Fig. 1).

**FIG. 2.**
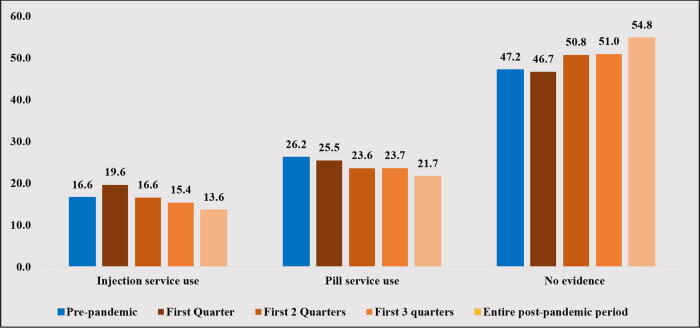
Changes in contraceptive method use after the COVID-19 pandemic among women enrolled in South Carolina Medicaid.

The pattern in method use was different for injections and pills (Fig. 2). The use of injectable contraceptives increased over the first quarter of the pandemic compared with the prepandemic level (19.6% vs. 16.6%, *p* < 0.001). The average use of injections exactly equaled the prepandemic level at the end of the first two quarters of the pandemic and consistently decreased over the rest of the pandemic periods. Conversely, the use of pills during the first quarter of the pandemic was lower than its prepandemic level (25.5% vs. 26.2%, *p* < 0.05). The use of pills consistently decreased over the rest of the pandemic periods. The percentage of people with no evidence of method use decreased during the first quarter of the pandemic and consistently increased during the rest of the pandemic periods (Fig. 2).

### Results from the ITSA

Results from the ITSA including the estimated coefficients and the 95% confidence intervals (CIs) are shown in Table 2. Positive baseline trends reflecting a strong Choose Well effect prepandemic were noted for IUD use (0.03 monthly percentage point change, 95% CI = 0.02, 0.04) and IUD insertion (0.01 monthly percentage point change, 95% CI = 0.01, 0.02). Significant increases in monthly trends during the pandemic period were noted for both IUD service use (0.05 monthly percentage point change, 95% CI = 0.04, 0.05) and IUD insertions (0.01 monthly percentage point change, 95% CI = 0.01, 0.012). A positive net change of 0.01 percentage points was noted for IUD service use. No net changes in trends comparing the prepandemic and pandemic periods were noted for IUD insertion.

**Table 2.** Changes in Contraception Use after the COVID-19 Pandemic Among Women Enrolled in South Carolina Medicaid: Interrupted Time-Series Regression Results (2017–2022)

	IUD service use	IUD insertion	Implant service use	Implant insertion
Change in level of method use, percentage points [95% CI]	−0.22***	−0.01	−0.46***	−0.47***
	[−0.37, −0.06]	[−0.12, 0.10]	[−0.61, −0.31]	[−058, −0.35]
Monthly prepandemic,	0.03***	0.01***	0.01***	0.01***
percentage points [95% CI]	[0.02, 0.04]	[0.01, 0.02]	[0.01, 0.02]	[0.01, 0.02]
Monthly pandemic,	0.05***	0.01***	0.05***	0.03***
percentage points [95% CI]	[0.04, 0.05]	[0.01, 0.012]	[0.04, 0.06]	[0.03, 0.04]
Change in trend,	0.01***	−0.003	0.04***	0.02***
percentage points [95% CI]	[0.01, 0.02]	[−0.01, 0.00]	[0.03, 0.05]	[0.01, 0.02]

The table reports coefficients from an interrupted time-series analysis in which the outcome variables (contraceptive use) are regressed on a baseline time trend (pre-COVID), a binary indicator variable for pandemic, and an interaction between the two. The monthly pandemic trend statistics were calculated using Stata’s “lincom” command, used to create linear combinations of the interaction term and the prepandemic trend regression coefficients.

***p < 0.001. **p < 0.05.

IUD, intrauterine device; CI, confidence interval.

Monthly trends for both implant service use and implant insertion were positive (0.01 monthly percentage point change, 95% CI = 0.01, 0.02) during the prepandemic Choose Well period. Both implant service use and insertion rebounded during the pandemic period with positive average monthly increases of 0.05 percentage points (95% CI = 0.04, 0.06) for implant service use and 0.03 percentage points for implant insertions (95% CI = 0.03, 0.04). Net positive gains in monthly trends for implant service use (0.04 percentage points per month) and implant insertion (0.02 percentage points per month) were noted.

## Discussion

This study examined if the trends in contraceptive method use among SC Medicaid beneficiaries during the latter half of Choose Well implementation were impacted by the COVID-19 pandemic. Our findings indicate that the impact of the COVID-19 pandemic on the utilization of contraceptive services among individuals enrolled in SC Medicaid was significant during the initial onset of the pandemic but was followed by a relatively rapid rebound, returning to prepandemic levels by the end of the year in 2020. Gains in IUD use observed among SC Medicaid beneficiaries during the initial 3 years of Choose Well implementation (prepandemic period) continued during the following 3 years (pandemic period), despite the initial decline in the early months of 2020. A significant positive trend in both IUDs and implant use among Medicaid beneficiaries occurred during the pandemic period beyond what would be expected had the prepandemic trends continued.

Significant decreases in the use of IUDs, implants, and pills were noted during the first quarter of the COVID-19 pandemic, spanning from March 2020 to May 2020. Decreases in IUD and implant method use during the initial months of the pandemic were more substantial than what was observed for pills. These findings are largely expected given early restrictions on in-person health service visits.^13^ Declines in the use of IUDs and implants persisted through the second quarter of the pandemic from June 2020 to August 2020. As 2020 drew to a close, however, the utilization of IUD slightly exceeded or matched prepandemic levels. The use of implant, in contrast, slightly lagged behind the prepandemic level. Overall, there was a small net change in IUD service use when examining the full duration of Choose Well’s pandemic period (March 2020 through December 2022) relative to Choose Well’s prepandemic period. These findings are positive and suggest that strong Choose Well trends in IUD use prepandemic continued during the pandemic period, despite the initial decline.

A notable and statistically significant positive trend in implant use occurred during the pandemic period beyond what would be expected had the prepandemic trends continued. This finding is largely attributed to the weak trend in implant use that existed before the pandemic period (over the first 3 years of Choose Well).^5^ The stronger trend in implant use in the second half of Choose Well (pandemic period) could reflect changes in method preferences of women over time or reflect further contributions of Choose Well to increasing access to these methods over the duration of the intervention.

Conversely, there was a substantial increase in the use of injectable contraceptives during the first quarter of the pandemic. This finding suggests that some women may have transitioned to injectable contraception as access to methods requiring in-person visits became a significant challenge. In the absence of IUD and implant methods, women seeking longer-acting methods may opt for injectable contraceptives given their longer duration of effectiveness than other hormonal methods and the potential for self-administration.^14^

Overall, our findings indicate that both providers and women may have reacted to the initial state of emergency by significantly altering their behavior, anticipating considerable disruptions to daily life and heightened health risks associated with COVID-19 transmission. It is possible that patients altered their contraceptive choices in order to avoid in-person visits and exposure to COVID-19. As restrictions eased, many women may have returned quickly to previous methods. Furthermore, the emergence and rapid adoption of telehealth services for contraceptive counseling may have also fostered a return to prepandemic method use.^15,16^ The expansion of online contraceptive counseling may have also provided benefits to those who had experienced difficulties in obtaining contraception previously.^17^ In addition, Choose Well could have served as a buffer and facilitated a rapid return to pre-COVID levels of method use.

Our analysis also revealed a significant increase in the number of observations indicating no contraception use in the pandemic period compared with prepandemic levels. This rise seems to be attributed to declines in injection and pill usage overall. Possible reasons for this trend include shifting preferences, such as fewer women opting for hormonal methods like the pill or injection due to concerns about side effects and instead choosing barrier methods or other nonhormonal options or opting not to use any method at all. In addition, changes in billing practices for injection and pill use over time may have affected the accuracy of data captured by Medicaid claims. It is imperative for future research to monitor these evolving trends closely and delve deeper into their underlying causes.

This study has some limitations. The study relied on Medicaid administrative claims data. While administrative data offer significant advantages, such as access to contraceptive receipt information for a large cohort of women, these data were not originally collected for research purposes. Consequently, there is the possibility of misclassifying Medicaid enrollment and service utilization. In addition, Medicaid claims data do not encompass information about contraceptives not reimbursed by Medicaid, including natural family planning methods, condoms, and other over-the-counter options. It is possible that some women in our study transitioned to nonreimbursed contraceptives during the pandemic. Furthermore, procedures involving IUDs and implants are likely to be more accurately recorded in claims data compared with injections and pills that require frequent provider engagement. While this strengthens the reporting accuracy for IUDs and implants, it may limit the extent to which conclusions can be drawn about other contraceptive methods. Another limitation of this study stems from our inability to distinguish between different types of services related to IUDs and implants. While the “IUD/implant service use” variables are good indicators of the overall demand for these services, they should be interpreted with caution as they encompass not only new use of IUDs/implants but also claims for consultations, follow-up visits for previously inserted IUDs/implants, and removals.

Lastly, this study specifically examined the impact of COVID-19 within the subset of women enrolled in the state’s Medicaid program. The extent to which contraceptive use patterns varied for women not enrolled in the state Medicaid program who are also receiving services fostered by Choose Well is not examined and should not be inferred.

## Conclusions

Despite the disruptions in the provision of contraceptive services during the early months of the pandemic, our findings indicate that contraceptive use within SC Medicaid recovered enough to average out initial declines over the months leading to the end of the initial funding period of Choose Well. While a short-term impact of the pandemic on contraceptive use was evident, these findings suggest that, overall, the pandemic had no significant impact on longer-term trends in IUD and implant method use among SC Medicaid beneficiaries. These findings provide further evidence of the impact of Choose Well in facilitating creating access to a wide range of contraceptive methods spanning a global pandemic.

While this study does not offer a comprehensive assessment of the pandemic’s overall impact on contraceptive receipt, it does provide valuable insights into the pandemic’s impact on access to contraceptive services among an important subgroup of women, particularly those who may be more vulnerable during health crises. The findings of this study provide important context for other studies that seek to evaluate the impact of the Choose Well initiative among women in the SC Medicaid program.

## Abbreviations Used

CI: Confidence Interval
COVID: Corona Virus Disease
FQHCs: Federally Qualified Health Centers
ITSA: Interrupted time series analysis
IUD: Intrauterine Device
SC: South Carolina
